# The Prognostic Value of Biomarkers Identified by [^18^F]FDG-PET/CT in Patients with High-Risk Melanoma Treated with Adjuvant Immunotherapy

**DOI:** 10.3390/diagnostics16010079

**Published:** 2025-12-25

**Authors:** Anne-Line Mayland Madsen, Oke Gerke, Christina H. Ruhlmann, Malene Grubbe Hildebrandt, Sambavy Nadaraja

**Affiliations:** 1 Department of Nuclear Medicine, Odense University Hospital, 5000 Odense, Denmark; anne-line.mayland.madsen@rsyd.dk (A.-L.M.M.); oke.gerke@rsyd.dk (O.G.);; 2 Department of Clinical Research, University of Southern Denmark, 5230 Odense, Denmark; 3 Department of Oncology, Odense University Hospital, 5000 Odense, Denmark

**Keywords:** adjuvant immunotherapy, anti-PD-1, biomarker, bone marrow-to-liver ratio, FDG-PET/CT, immune-related adverse events, melanoma, molecular imaging, spleen-to-liver ratio

## Abstract

**Background**: Adjuvant anti-PD-1 therapy improves recurrence-free survival (RFS) in high-risk melanoma, but many patients experience adverse events. 2-deoxy-2-[^18^F]fluoro-D-glucose positron emission tomography with computed tomography [^18^F]FDG-PET/CT has been proposed to identify biomarkers that may predict outcome of treatment. **Objectives**: The aim of this register-based study was to investigate the prognostic value of immune-related adverse events (irAEs), spleen-to-liver ratio (SLR), and bone marrow-to-liver ratio (BLR), detected by [^18^F]FDG-PET/CT. **Methods**: This retrospective, register-based cohort study included 122 patients with radically resected stage III–IV melanoma treated with adjuvant anti-PD-1. Patient data were extracted from a Danish register, and measurements for SLR and BLR were made using an AI model. Cox regression models were made on irAEs and BLR to assess associations with RFS and overall survival (OS). **Results**: Over half of the patients experienced recurrence, and one quarter died during follow-up of 4 ¾ years. Seventy-four percent exhibited at least one PET-detected irAE. This study found no association between irAEs and OS. Regarding RFS, our findings suggest an increased risk of recurrence for the presence of irAEs within the first 1.5 years of follow-up (HR: 2.93, CI: 1.10–7.84, *p* = 0.032). BLR and SLR were not associated with RFS or OS in multivariable models. **Conclusions**: This study did not confirm the findings of a positive association between irAEs and survival found in previous studies. PET-detected irAEs were common in the study population, but did not predict OS, while early-onset irAEs were linked to increased recurrence risk. Neither SLR nor BLR demonstrated prognostic value. Further research is needed to clarify the clinical utility of PET-derived biomarkers, especially in the adjuvant setting.

## 1. Introduction

The introduction of adjuvant treatment with immune checkpoint inhibitors (ICIs), such as programmed cell death protein 1 monoclonal antibodies (anti-PD-1), has improved recurrence-free survival (RFS) in high-risk melanoma patients [1,2,3,4], but not all patients benefit from the treatment. In the setting of patients undergoing adjuvant treatment, patients are considered melanoma-free, as the visible tumor tissue has been surgically removed. Therefore, an analysis of response to anti-PD-1 in this patient setting is not possible. However, a recent study in patients with resected stage III melanoma receiving nivolumab as adjuvant treatment reported that number needed to treat (NNT) at four years was 4.2 for RFS and 8.5 for overall survival (OS) [5]. This means that a substantial number of patients receiving adjuvant treatment with nivolumab obtain no benefit from the treatment, indicating a need for tools that aid in the distinction of patients who benefit from the treatment from those who do not. This has both an economic and a health-related value in avoiding unnecessary toxicity. In recent years, multiple studies have proposed 2-deoxy-2-[^18^F]fluoro-D-glucose positron emission tomography with computed tomography ([^18^F]FDG-PET/CT) as a potential tool for identifying prognostic biomarkers to distinguish those who benefit from treatment from those who do not [6]. According to the European Society for Medical Oncology (ESMO) guidelines for melanoma, the follow-up of high-risk patients may include imaging exams such as the ultrasound (US) of lymph nodes, computed tomography (CT), or whole-body positron emission tomography (PET) [7]. In Denmark, recommendations for patients with resected stage IIIA–IV melanoma receiving adjuvant treatment for up to one year are [^18^F]FDG-PET/CT scans every three months while treatment is ongoing [8]. The non-invasive nature of [^18^F]FDG-PET/CT further underlines its advantage.

Previous studies have proposed that [^18^F]FDG-PET/CT-detectable biomarkers, such as immune-related adverse events (irAEs), the spleen-to-liver ratio (SLR), and the bone marrow-to-liver ratio (BLR), are potential prognosticators for patient outcome [6,9,10,11,12,13,14,15,16]. In the case of irAEs, studies have reported that approximately 14% of patients treated with adjuvant anti-PD-1 experience grade 3–5 irAEs. Although grade 5 events are exceedingly rare, severe irAEs often lead to treatment discontinuation [2,17]. Many studies have reported that the occurrence of irAEs is a positive prognostic factor for patient outcome [6,18,19,20,21]. Furthermore, it has been demonstrated that [^18^F]FDG-PET/CT can aid in the detection of irAEs, potentially even before they present with clinically detectable symptoms [21,22,23,24]. A recent study by our group has concluded that [^18^F]FDG-PET/CT can assist in the detection of irAEs with clinically acceptable accuracy [25]. Despite this potential, studies on the prognostic value of irAEs identified on [^18^F]FDG-PET/CT are, to the best of our knowledge, few and either include small populations or are restricted to only one type of [^18^F]FDG-PET/CT-identified irAE [9,21,26,27,28].

In recent years, an increase in the ratio of the standardized uptake value (SUV) in hematopoietic tissues (such as the spleen and bone marrow) and the SUV in the liver (SLR and BLR, respectively) has been investigated as a negative prognostic biomarker for treatment response both in the pretreatment setting and over time during treatment, though with varying results [6,9,10,11,12,13,14,15,16]. Importantly for the implementation of SLR and BLR as prognostic biomarkers, a 2021 study by Prigent et al. [9] reported good interobserver reproducibility of the SLR and BLR measurements. However, the emergence of studies on SLR and BLR as prognostic biomarkers is relatively recent, and, to the best of our knowledge, there are currently no studies on the prognostic value of pretreatment SLR and BLR measurements in the setting of melanoma patients receiving adjuvant treatment.

The aim of this register-based study from Denmark, focusing on high-risk melanoma patients treated with adjuvant anti-PD-1, was to investigate the prognostic value of immune-related adverse events, the spleen-to-liver ratio, and the bone marrow-to-liver ratio detected by [^18^F]FDG-PET/CT. Specifically, we examined the impact on recurrence-free survival (RFS), and overall survival (OS).

## 2. Materials and Methods

In this register-based cohort study we investigated the prognostic value of irAEs, SLR, and BLR identified with [^18^F]FDG-PET/CT in patients with resected high-risk (stage III–IV) melanoma treated with adjuvant immunotherapy. The population of this study was first included in a previous study by our group [29]. Patient data were extracted from the Danish Metastatic Melanoma Database (DAMMED) [30] on the 12th of May 2025 and from another previous study by our group on the same population [25]. The previous two studies on this patient group investigated the diagnostic accuracy and clinical impact of [^18^F]FDG -PET/CT follow-up [29] and organ-specific accuracy of [^18^F]FDG-PET/CT in identifying immune-related adverse events in this patient group [25].

### 2.1. Ethics

The retrospective, register-based cohort study did not influence the treatment of the patients included. The study has been recorded in the General Data Protection Regulation (GDPR) register of the Region of Southern Denmark (JR nr.: 20/59961). Written consent to participate in melanoma-related research was obtained from the patients before registration in the DAMMED [30].

### 2.2. Study Population

We included high-risk melanoma patients from the region of Southern Denmark who had undergone radical resection, at least one follow-up scan, and received at least one dose of anti-PD-1 at Odense University Hospital between November 2018 and February 2021. A total of 124 patients were included in the population. For more in-depth details, refer to our previous study [29]. The inclusion criteria of the current study were patients from the region of Southern Denmark with radically resected high-risk melanoma, a pretreatment (baseline) scan, registration of the date of treatment initiation, at least one follow-up scan, and at least one dose of anti-PD-1. Patient data regarding treatment, stage, B-Raf proto-oncogene (BRAF) mutation status, performance status (PS), blood lactate dehydrogenase (LDH) level, age at first treatment, date for first treatment, date for recurrence or last visit without recurrence, date of death or last visit alive, and cause of death were all collected from the DAMMED [30]. Data regarding comorbidities were collected from the second of the previous studies by this group, published in 2024 [25]. Data from the patients’ medical records were collected and then stored using Research Electronic Data Capture (REDCap) tools hosted at OPEN, Odense, Denmark [31,32]. For more in-depth details, refer to our 2024 study [25].

### 2.3. irAEs on [^18^F]FDG-PET/CT

The [^18^F]FDG-PET/CT scans were conducted in accordance with the Danish national guidelines [8] for follow-up of melanoma patients undergoing adjuvant treatment and performed at one of three locations: Odense University Hospital, Odense; Hospital of South West Jutland, Esbjerg; or Lillebælt Hospital, Vejle. The [^18^F]FDG-PET/CT scans were performed <four weeks before treatment (baseline) and every three months during treatment. Protocols for each of the three locations can be seen in the appendix. The irAEs on the [^18^F]FDG-PET/CT scans were identified for the 2024 study by this group as follows: The scans were analyzed by three nuclear medicine specialists who used a standardized protocol and were blinded to patients’ medical records. First, the baseline scan, from before initiation of treatment, was assessed for physiologic FDG uptake. Subsequently, each follow-up scan was compared with the respective previous scan with regard to organ-related FDG uptake to note whether there was no change, the appearance of new FDG uptake, the appearance of increased FDG uptake, or the appearance of decreased FDG uptake. These were all determined for each of the following organs/organ systems: the pituitary gland, the thyroid, the lungs, the heart, the gastrointestinal (GI) tract, the joints, the muscle, the skin, and the lymph nodes. For more in-depth details on scan interpretations, refer to our 2024 study [25]. In this study, organ-related FDG uptake was considered an irAE every time it appeared and when it had increased but had neither previously been marked as new nor increased. The time of the [^18^F]FDG-PET/CT scan when an irAE first appeared was recorded for each patient for each of the above-mentioned organ locations and for irAEs in general.

### 2.4. SLR and BLR on [^18^F]FDG-PET/CT

The measurements for SLR and BLR were made with the assistance of the Research Consortium for Medical Image Analysis (RECOMIA) at Lund, Sweden, a not-for-profit organization with the objective of promoting research in the fields of artificial intelligence and medical imaging [33]. Our research group has an established collaboration with RECOMIA on several studies, both previous and ongoing. The Swedish organization is independent of Odense University Hospital and the Danish health care system in general and has no stakes in the outcomes of the studies. RECOMIA provided the measurements of BLR and SLR.

### 2.5. SLR

A convolutional neural network (CNN) developed by RECOMIA called Organ Finder was used to segment the liver and the spleen and then use that segmentation to obtain the SUV_mean_ of both the liver and the spleen (^liver^SUV_mean_ and ^spleen^SUV_mean_, respectively) [34]. We then calculated the SLR, as follows:

SLR=SUVmeanspleenSUVmeanliver

An SLR higher than the median SLR was considered abnormal.

### 2.6. BLR

Sadik, M. et al. [35] trained and tested an artificial intelligence (AI) model for the detection of focal skeleton/bone marrow uptake (BMU) in their study. In short, they used a CNN to identify all of the bones. Then, they created a marrow region by excluding the edges from each individual bone, simulating where the bone marrow sites (the amount excluded depended on the bone in question). They grouped the bones into two parts: “spine” (including the vertebrae, sacrum, and coccyx as well as regions in the hipbones within 50 mm from these locations) and “other bones” (including the humeri, scapulae, clavicles, ribs, sternum, femora, and the remaining parts of the hipbones). For each group, the focal SUVs were measured.

In this study, we used the same model of segmentation of the bone marrow to derive the AI-model based mean SUV of the total bone marrow (^BM^SUV_mean_). We then calculated the BLR, as follows:

BLR=SUVmeanBMSUVmeanliver

A BLR higher than the median BLR was considered abnormal.

The calculations were based on information that were obtainable from the previous studies regarding this subject [6,9,10,11,12,13,14,15,16].

### 2.7. Statistical Analyses

Descriptive statistics comprised median (range) for continuous variables and frequencies with respective percentages for categorical variables.

The occurrence of an irAE at month 3, 6, 9, 12, or 16 was a time-varying factor. As such, to mitigate potential immortal time bias, it was coded 0 before an irAE had been identified and a value of 1 thereafter. Patients who did not show any signs of irAEs had a value of 0 for the entirety of the follow-up period. Follow-up started at first treatment of the patient. Time-to-event analyses consisted of extended Kaplan–Meier plots [36] and Cox proportional hazards regression models. OS and RFS were regressed on stage, BRAF mutation status, PS, LDH level, age, gender, relevant comorbidities, and the time varying covariate irAE (yes/no). Comorbidities noted as relevant were other cancers, both previous and ongoing, and immune-related conditions like psoriasis, rheumatoid arthritis, and sarcoidosis. We reported hazard ratios (HRs), their 95% confidence intervals (CIs), and *p*-values. We assessed the proportional hazards assumption with log–log plots and goodness-of fit tests. The interdependence of explanatory (i.e., independent) variables in regression modelling was evaluated with variance inflation factors. When the data suggested a violation of the proportional hazards assumption, we decided post-hoc to fit an extension of the Cox proportional hazards regression model and derived distinct HRs for the group difference for two observational periods that were characterized by a follow-up time point where the Kaplan–Meier curves crossed. To this end, we employed so-called heaviside functions [37].

The above time-to-event analyses were analogously repeated where SLR and BLR replaced irAE as explanatory factors. SLR, BLR, and irAE were pairwise highly correlated; therefore, they exclude each other in multivariable modeling.

The level of statistical significance was 5% (two-sided). All analyses were undertaken with STATA/MP 17 (StataCorp, College Station, TX, USA).

## 3. Results

### 3.1. Patient Characteristics

A total of 124 patients were considered for inclusion [29]. Based on the predefined eligibility criteria, two patients were excluded: one due to the absence of a baseline scan, and one owing to missing information on the treatment initiation date. Of the 122 patients, 35 (28.7%) died during follow-up, of whom two (1.6%) did not experience recurrence. Seventy patients (57.4%) experienced recurrence, of whom 33 (27%) died during follow-up. Accordingly, 72 events (59%) were observed for RFS.

The median follow-up period for the 122 patients was 4 ¾ years (that is 1723 days, range 117–2265 days).

One patient had missing data on baseline LDH level and was therefore excluded from the multivariate analyses, resulting in 121 patients available for multivariate analyses. Baseline characteristics are presented in Table 1.

**Figure 1:**
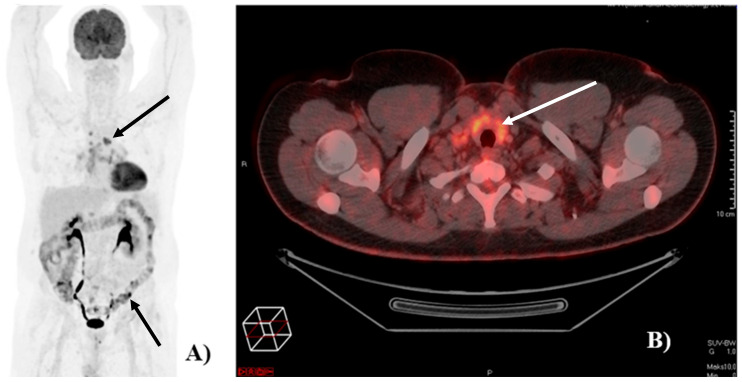
Image of [18F]FDG-PET/CT-detected immune-related adverse events (irAEs). (**A**) Example of a patient with [18F]FDG-PET/CT-detected irAEs in the colon and in mediastinal lymph nodes (black arrows). (**B**) Example of a patient with [18F]FDG-PET/CT detected irAE in the thyroid (white arrow).

Of the 121 patients, 90 (74%) had at least one [^18^F]FDG-PET/CT detected irAE. Image examples of irAEs detected on [^18^F]FDG-PET/CT are shown in Figure 1.

All patients received nivolumab; however, one switched to pembrolizumab due to an allergic reaction. Of the 90 patients with [^18^F]FDG-PET/CT-detected irAEs, 48 patients (53%) had more than one. As 6 patients had two irAEs in the same organ/organ system (5 in the GI tract and 1 in joints), the total number of irAEs identified on [^18^F]FDG-PET/CT was 165. The majority of irAEs (56%) were detected at the 3-month follow-up scan (Table 2).

For the SLR and BLR analyses, one patient was excluded due to technical issues from our side, related to the upload of the baseline scan. Consequently, 121 patients were included in the univariate analyses and 120 in the multivariate analyses.

### 3.2. Overall Survival

**Figure 2:**
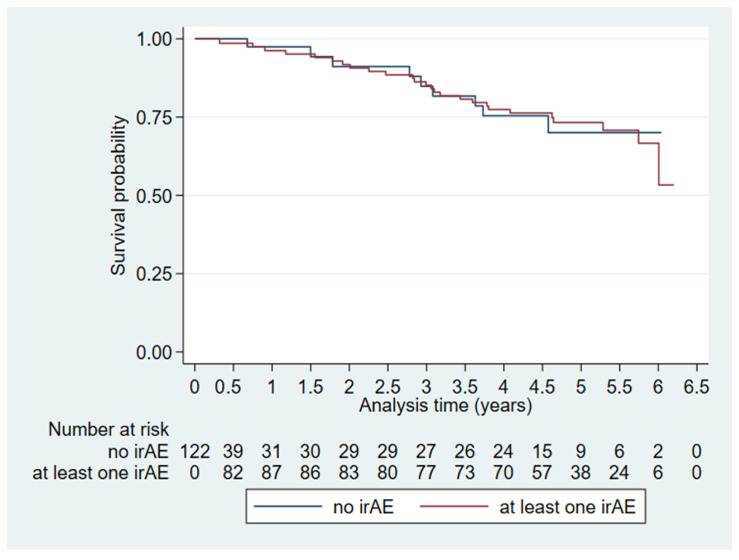
Extended Kaplan–Meier plot visualizing time-dependent immune-related adverse events (irAEs) on overall survival (*n* = 122).

The multivariate OS analysis of irAEs (Table 3, Model 1) revealed no difference between those who had irAEs on [^18^F]FDG-PET/CT and those who did not. This was the case when looking purely at the presence of irAEs (HR: 0.74, CI: 0.12–4.45, *p *= 0.74) and when looking at irAEs as a time-varying variate (HR: 1.001, CI: 0.999–1.002, *p *= 0.54). Figure 2 shows a Kaplan–Meier plot for visual representation of the effect of irAEs on overall survival over time.

**Figure A1:**
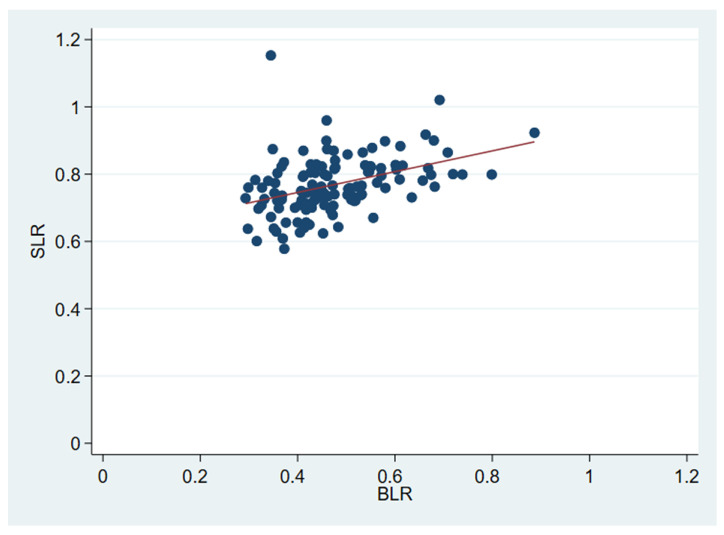
Scatterplot of BLR and SLR visualizing positive correlation. The red line indicates the least-squares linear regression of SLR on BLR.

**Figure A2:**
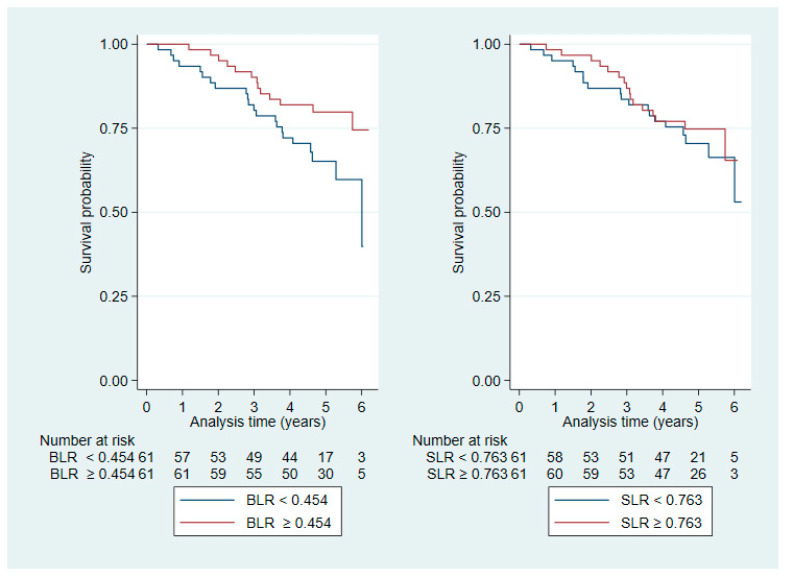
Kaplan–Meier plots stratified by median values for BLR (**left**) and SLR (**right**) for OS.

**Figure A3:**
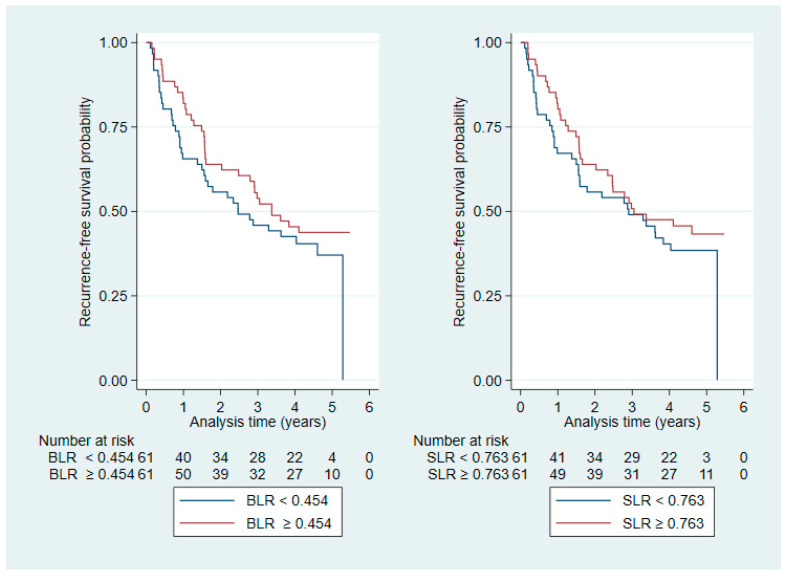
Kaplan–Meier plots stratified by median values for BLR (**left**) and SLR (**right**) for RFS.

BLR and SLR were positively correlated (Pearson’s r: 0.4, *p* < 0.0001; Figure A1 in Appendix A), and Kaplan–Meier plots stratified by median values (BLR: 0.454, SLR: 0.763) suggested a stronger visual effect of BLR than SLR on OS and RFS (Figure A2 and Figure A3, respectively, in Appendix A). Therefore, we investigated BLR in multivariable modelling as a potential explanatory factor (Table 3, Model 2). The results from the multivariate OS analysis of BLR revealed no difference between those who had a BLR > 0.454 and those who did not (HR: 0.012, CI: 0.0001–1.07, *p *= 0.054).

### 3.3. Recurrence-Free Survival

**Figure 3:**
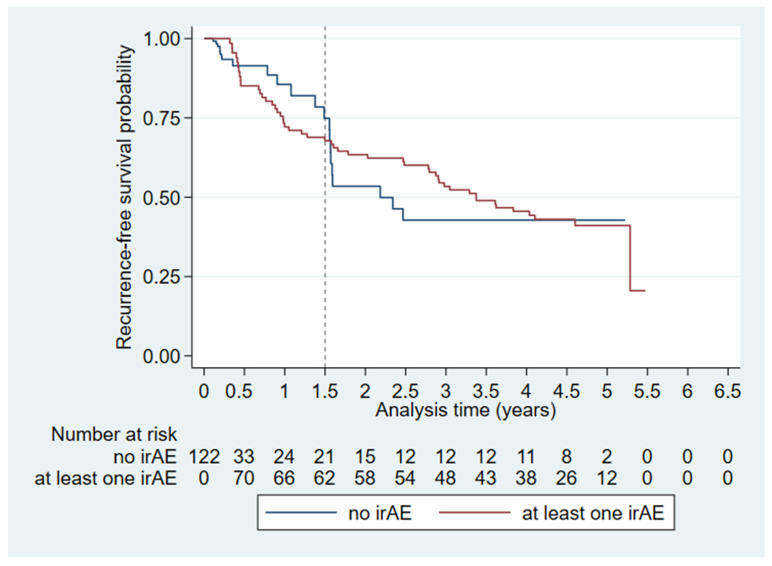
Extended Kaplan–Meier plot visualizing time-dependent irAEs on recurrence-free survival (*n* = 122). The dashed line indicates the split of the analysis time at 1.5 years where the Kaplan-Meier curves cross.

When analyzing the irAEs we found that the Kaplan–Meier survival curves for patients who experienced irAEs and those who did not crossed after 1.5 years of follow-up (Figure 3) whereby the proportional hazards assumption did not hold. We assessed the time period of up to 1.5 years follow-up and 1.5 years of follow-up or more individually (Table 4, Model 3). Presence of irAEs within the first 1.5 years of follow-up was associated with an increased risk of recurrence (HR: 2.93, CI: 1.10–7.84, *p* = 0.032). In multivariate irAE RFS-analysis (Table 4, Model 3), 71 of 121 patients had an event, and the distribution of events between the <1.5 years group and the ≥1.5 years group were 38 events and 33 events, respectively.

We investigated BLR (and by extension SLR) in multivariable modelling as a potential explanatory factor (Table 4, Model 4). The multivariate RFS analysis of BLR revealed no difference between those who had a BLR > 0.454 and those who did not (HR: 0.19, CI: 0.013–2.65, *p* = 0.22).

Of note is the statistically significant reduced HR in patients with comorbidities in both models (Model 3; HR: 0.50, CI: 0.28–0.89, *p* = 0.018 and Model 4; HR: 0.48, CI: 0.26–0.87, *p* = 0.015).

## 4. Discussion

### 4.1. Summary of Main Findings

This study investigated the prognostic value of immune-related adverse events, spleen-to-liver ratio, and bone marrow-to-liver ratio detected by or measured on [^18^F]FDG-PET/CT in a population of patients with resected high-risk melanoma, treated with adjuvant anti-PD-1. Of the 121 patients included in the multivariate analysis of irAEs, 90 (74%) had at least one irAE that was identified on [^18^F]FDG-PET/CT. The majority of irAEs (56%) were observed at the 3-month follow-up scan. We found that the occurrence of irAEs within the first 1.5 years of follow-up was associated with an increased risk of recurrence in the RFS analysis.

Regarding BLR (and by extension SLR), no association was observed between FDG uptake ratios higher than the median, neither for OS nor RFS.

### 4.2. Strengths and Limitations

As this was a retrospective register-based study, the data collected reflected clinical practice, providing an accurate presentation of treatments and outcomes in the adjuvant setting. The prospective inclusion of all patients with high-risk melanoma in the Danish register minimized the risk of selection bias as all but one patient meeting the inclusion criteria were included in the irAE analyses and all but two in the BLR analyses. One of the greatest strengths of this study was the use of a statistical model that allocated a patient’s irAE-free and irAE-persisting time intervals to the respective groups. Thereby, we assessed survival unbiased in terms of irAE onset, and we adjusted the analyses for possible confounders. Another strength lies in the ability to segment whole organs, instead of having to use volumes of interest (VOIs) for our BLR/SLR analyses.

A limitation of our study lies in the use of retrospectively analyzed [^18^F]FDG-PET/CT scans to detect irAEs. Even though [^18^F]FDG-PET/CT can detect irAEs with a clinically acceptable diagnostic accuracy, false positive findings were the most significant source of detection error in our previous study, which means that we overestimate the occurrence of irAEs. At the same time, we might miss some irAEs as they could have occurred and been treated in the time between scans. Strengths and limitations of using [^18^F]FDG-PET/CT to detect irAEs have been discussed in further detail in our previous study [25]. Another point worth mentioning is that we had to split up the “Presence of irAEs” into the two time intervals in the RFS analysis. This prohibits one from making a conclusion for irAEs throughout the entire follow-up period. These findings should be taken with a grain of salt and the clinical relevance of this put to question.

Compared with previous investigations of the prognostic value of [^18^F]FDG-PET/CT-detected irAEs, our study included the largest number of patients [9,21,26,27,28], and, regarding BLR and SLR, our study was the first to explore the prognostic values in the setting of adjuvant treatment of melanoma patients [6,9,10,11,12,13,14,15,16].

### 4.3. Possible Explanations for Findings

As patients in the setting of adjuvant treatment are considered melanoma-free and are expected to have better survival than their non-resectable counterparts, a median follow-up time of 4 ¾ years might not be long enough to detect differences in survival. However, with 35 events in the OS and 72 events in the RFS analyses in a population of 122 patients (29% and 59% respectively), it is unlikely to be the most significant contributing factor to our findings. Of the previous studies of irAEs detected on [^18^F]FDG-PET/CT [9,21,26,27,28], findings vary between no statistical significance and statistically significant positive prognostic effects of their respective outcomes, but our study is the first to find an increased risk of a negative outcome. One possible explanation for these unclear findings is the lack of proper guidelines to define what indicates an irAE on [^18^F]FDG-PET/CT, which may result in misclassification of [^18^F]FDG-PET/CT-detected irAEs and contribute to between-study variability. Another possibility is that the irAE survival advantage in some studies [18] stems from assigning a patient to the irAE group with both irAE-free and irAE-persisting time intervals [36], thereby overestimating the survival time in irAE patients compared with irAE-free patients.

As lymphoid organs, both the bone marrow and the spleen play a role when it comes to inflammation, and high splenic FDG uptake has been observed in patients with infections as well as patients with cancers associated with an inflammatory state [16]. As of this study, there is still no clearly agreed-upon explanation for the findings in previous studies. However, one theory suggests that increased splenic activity in cancer patients may be due to an increase in myeloid-derived suppressor cells (MDSCs), which limit productive immune responses against tumors (that is immunosuppressive MDSCs) [38]. If this holds true, it could explain why we do not find SLR or BLR to be prognostic factors when examining a population of fully resected patients, where there is no tumor to respond to.

### 4.4. Perspective

The use of [^18^F]FDG-PET/CT-detected irAEs as a biomarker was not meant to stand on its own, but rather to be one of the different factors clinicians use to evaluate if a patient is experiencing irAEs. It would provide a better reflection of real-world use if the [^18^F]FDG-PET/CT-detected irAEs were compared with other indicators of clinically significant irAEs, such as patient-reported irAEs, steroid treatment, and premature discontinuation of anti-PD-1 therapy. As findings of studies on irAEs detected on [^18^F]FDG-PET/CT vary, future studies are indicated. We propose that they also include the aforementioned parameters to evaluate a more representative value of irAE as a biomarker in clinical practice.

If high FDG uptake in lymphoid organs occurs in response to the presence of cancer (as mentioned above) and we found no association between having a BLR higher than the median and OS or RFS, further studies in populations of fully resected patients are not indicated.

## 5. Conclusions

In this study on patients with high-risk melanoma treated with adjuvant immunotherapy, six out of ten experienced recurrence, and one quarter died during a median follow-up of nearly five years. Immune-related adverse events were common, with almost three-quarters of the patients showing at least one irAE on [^18^F]FDG-PET/CT follow-up scans, and half presented with more than one event.

Contrary to previous studies, we found no association between irAEs and survival. However, irAEs occurring within the first 1.5 years of follow-up were associated with an increased risk of recurrence.

Neither bone marrow-to-liver nor spleen-to-liver FDG uptake ratios above the median were associated with overall or recurrence-free survival.

## Figures and Tables

**Table 1:** Patient characteristics at baseline (*n* = 122). Descriptive statistics show median (minimum–maximum) for continuous variables and frequency tables with respective percentages for categorical variables.

Variable	Factor Level	Descriptive Statistics
Age (in years)		62 (18–84)
Sex	Female	48 (39%)
	Male	74 (61%)
Performance status	0	109 (89%)
	1	13 (11%)
Comorbidities	Yes	38 (31%)
	No	84 (69%)
Stage	IIIA	18 (15%)
	IIIB, IIIC, and IIID	89 (73%)
	IV	15 (12%)
BRAF status	Wildtype	44 (36%)
	Mutation	36 (30%)
	Not tested	42 (34%)
Lactate dehydrogenase	≤Median	63 (52)
	>Median	58 (48)
BLR		0.454 (0.293–0.887)
SLR		0.763 (0.578–1.153)

Abbreviation: BRAF, B-Raf proto-oncogene.

**Table 2:** Overview of *n* = 165 irAEs.

Variable	Factor Level	Descriptive Statistics
Number of patients	With irAE	90 (74%)
	Without irAE	32 (26%)
Location	Heart	5 (3%)
	Pituitary	8 (5%)
	Muscles	12 (7%)
	Skin	13 (8%)
	Lungs	15 (9%)
	Thyroid	17 (10%)
	Joints	23 (14%)
	Lymph nodes	33 (20%)
	Gastrointestinal	39 (24%)
Time point of irAE	3 months	92 (56%)
	6 months	39 (24%)
	9 months	15 (9%)
	12 months	17 (10%)
	Extra scans	2 (1%)

Abbreviation: irAE, immune-related adverse event.

**Table 3:** Extended Cox proportional hazards regression on overall survival. Model 1: with irAEs (*n* = 121). Model 2: with BLR (*n* = 120).

Variable	Factor Level	HR	Model 1 95% CI	*p*-Value	HR	Model 2 95% CI	*p*-Value
LDH (ref.: LDH ≤ median)	LDH > median	0.97	0.46–2.04	0.93	0.99	0.47–2.06	0.97
Stage (ref.: IIIA)	IIIB, IIIC, IIID	0.82	0.24–2.80	0.75	1.08	0.32–3.72	0.90
	IV	1.25	0.29–5.38	0.77	1.87	0.40–8.61	0.42
BRAF status (ref.: Wildtype)	Mutation	0.58	0.25–1.31	0.19	0.61	0.25–1.48	0.28
	Not tested	0.20	0.07–0.63	**0.006**	0.27	0.09–0.84	**0.023**
Age		1.01	0.98–1.04	0.71	0.99	0.96–1.02	0.63
Sex (ref.: male)	Female	1.12	0.55–2.31	0.76	1.25	0.58–2.67	0.57
Performance status (ref.: 0)	Yes	1.01	0.33–3.11	0.98	0.97	0.27–3.50	0.96
Comorbidities (ref.: no)	Yes	0.85	0.40–1.82	0.67	0.81	0.37–1.76	0.59
Presence of irAEs (ref.: no)	Yes	0.74	0.12–4.45	0.74			
Time-varying irAEs (ref.: no)	Yes	1.001	0.999–1.002	0.54			
BLR					0.012	0.0001–1.07	0.054

Bold print indicates *p*-values below the significance level of 5%. Abbreviations: BLR, bone marrow-to-liver ratio. BRAF, B-Raf proto-oncogene. CI, confidence interval. HR, hazard ratio. irAEs, immune-related adverse events. LDH, Lactate dehydrogenase. Ref., reference.

**Table 4:** Extended Cox proportional hazards regression on recurrence-free survival. Model 3: with irAEs (*n* = 121). Model 4: with BLR (*n* = 120).

Variable	Factor Level	HR	Model 3 95% CI	*p*-Value	HR	Model 4 95% CI	*p*-Value
LDH (ref.: LDH ≤ median)	LDH > median	1.19	0.72–1.99	0.50	1.29	0.77–2.16	0.34
Stage (ref.: IIIA)	IIIB, IIIC, IIID	1.44	0.59–3.51	0.43	1.85	0.76–4.50	0.18
	IV	2.33	0.78–6.99	0.13	3.28	1.03–10.5	**0.045**
BRAF status (ref.: Wildtype)	Mutation	0.85	0.50–1.46	0.56	0.90	0.51–1.60	0.73
	Not tested	0.09	0.03–0.22	**<0.0001**	0.10	0.04–0.26	**<0.0001**
Age		1.00	0.98–1.02	0.94	1.00	0.98–1.02	0.64
Sex (ref.: male)	Female	1.27	0.77–2.10	0.35	1.36	0.81–2.29	0.25
Performance status (ref.: 0)	Yes	0.59	0.24–1.44	0.25	0.70	0.25–1.91	0.48
Comorbidities (ref.: no)	Yes	0.50	0.28–0.89	**0.018**	0.48	0.26–0.87	**0.015**
Time-varying irAEs	0–1.5 years f.u.	2.93	1.10–7.84	**0.032**			
	≥1.5 years f.u.	0.86	0.38–1.96	0.72			
BLR					0.19	0.01–2.65	0.22

Bold print indicates *p*-values below the significance level of 5%. Abbreviations: BLR, bone marrow-to-liver ratio. BRAF, B-Raf proto-oncogene. CI, confidence interval. F.u., follow-up. HR, hazard ratio. irAEs, immune-related adverse events. LDH, Lactate dehydrogenase. Ref., reference.

## Data Availability

The data of this study cannot be shared due to legal restrictions.

## Abbreviations

The following abbreviations are used in this manuscript:

ICI	Immune checkpoint inhibitor
anti-PD-1	Programmed cell death protein 1 monoclonal antibodies
RFS	Recurrence-free survival
NNT	Number needed to treat
OS	Overall survival
[^18^F]FDG-PET/CT	2-deoxy-2-[^18^F]fluoro-D-glucose positron emission tomography with computed tomography
ESMO	European Society for Medical Oncology
US	Ultrasound
CT	Computed tomography
PET	Positron emission tomography
irAE	Immune-related adverse event
SLR	Spleen-to-liver ratio
BLR	Bone marrow-to-liver ratio
SUV	Standardized uptake value
DAMMED	Danish Metastatic Melanoma Database
GDPR	General Data Protection Regulation
BRAF	B-Raf proto-oncogene
PS	Performance status
LDH	Lactate dehydrogenase
REDCap	Research electronic data capture
GI	Gastrointestinal
RECOMIA	Research Consortium for Medical Image Analysis
CNN	Convolutional neural network
AI	Artificial intelligence
BMU	Bone marrow uptake
^liver^SUV_mean_	Mean standardized uptake value of the liver
^spleen^SUV_mean_	Mean standardized uptake value of the spleen
^BM^SUV_mean_	Mean standardized uptake value of the total bone marrow
HR	Hazard ratio
CI	Confidence intervals
VOI	Volumes of interest
MDSC	Myeloid-derived suppressor cell

## Appendix A

### Appendix A.1. [^18^F]FDG-PET/CT Scan Protocol at Odense University Hospital

PET/CT data were acquired on a GE Discovery 710 PET/CT scanner. The PET scan was performed using a standard whole-body acquisition protocol with an acquisition time of 2½ min per bed position. The scan field of view was 70 cm. PET data were reconstructed into transaxial slices with a matrix size of 256 × 256 (pixel size 2.74 mm) and a slice thickness of 3.75 mm using iterative 3D OS-EM (3 iterations, 24 subsets) with corrections for time-of-flight (GE VPFX) and point-spread blurring (GE SharpIR). Attenuation correction was based on a dedicated ultra-low-dose helical CT attenuation correction scan. A helical diagnostic CT scan was acquired after the PET scan with intravenous contrast (ULRAVIST 370 I/mL) using a standard CT protocol with a scan field of view of 70 cm. Data were reconstructed with a standard filter into transaxial slices with a field of view of 50 cm, matrix size of 512 × 512 (pixel size 0.98 mm) and a slice thickness of 3.75 mm. Analysis of CT, PET, and fused PET/CT data was done on a GE Advantage Workstation v. 4.4. The CT scan was evaluated by a radiologist and the PET scan by a nuclear medicine specialist.

### Appendix A.2. [^18^F]FDG-PET/CT Scan Protocol at the Hospital of South West Jutland, Esbjerg

PET/CT data were acquired on either a GE Discovery 710 PET/CT scanner (710) or GE Discovery MI (MI) PET/CT scanner with 25 cm axial field of view. The PET scan was performed using a standard whole-body acquisition protocol with an acquisition time of either 2½ min per bed position (710) or 1½ min per bed (MI). The scan field of view was 70 cm. PET data were reconstructed into transaxial slices with a matrix size of 256 × 256 (pixel size 2.74 mm) and a slice thickness of either 3.27 mm (710) or 2.8 mm (MI) using a block sequential regularized expectation maximization algorithm (GE Q.Clear) with a penalizing factor of 350 and using time-of-flight. Attenuation correction was based on a dedicated ultra-low dose helical CT attenuation correction scan. According to regional protocol, the initial scan included the acquisition of a helical diagnostic CT scan after the PET scan with in-vivo contrast (ultravist 370 I/mL), whereas subsequent control scans included the acquisition of a low-dose CT scan only. Both scans were performed using a standard CT protocol with a scan field of view of 70 cm. Data were reconstructed with a standard filter into transaxial slices with a field of view of 50 cm, matrix size of 512 × 512 (pixel size 0.98 mm) and a slice thickness of 3.75 mm. Initially, the scan field was a modified whole-body protocol covering from vertex cranii to mid-femoral unless indicated otherwise by disease location, but from august 2020 a complete whole-body protocol from vertex cranii to the toes was implemented routinely. The CT scan was described by a radiologist and the PET scan by a nuclear medicine specialist.

### Appendix A.3. [^18^F]FDG-PET/CT Scan Protocol at Lillebælt Hospital, Vejle

[^18^F]FDG-PET/CT scans were performed in Vejle on four PET/CT scanners with a scan field from vertex cranii to ankle level (165 cm) and an equivalent acquisition time of 2 min per bed position at truncus and 1.5 min per bed over the legs. A low dose helical CT scan was performed for attenuation correction, localization, and characterization of lesions. The acquisition and reconstruction parameters varied across scanner types as described below. When requested, a diagnostic contrast enhanced CT was performed on the same day as a separate procedure. Analysis of PET and fused PET/CT data was undertaken on a Hermes application v. 2.3.0 and diagnostic CT on an easyViz PACS system v 8.1.0. The PET/CT scan was assessed by a nuclear medicine specialist and a radiologist, side-by-side.

Siemens Biograph Vision PET/CT scanner: Transaxial scan field of view of 78 cm with PET data reconstructed in a matrix size of 220 × 220 (pixel size 3.5 mm) and a slice thickness of 4 mm using iterative 3D OS-EM (4 iterations, 5 subsets) with corrections for time-of-flight and point-spread function (TrueX/UltraHD PET). CT data were reconstructed with a medium smooth filter into transaxial slices with a field of view of 78 cm, matrix size of 512 × 512 (pixel size 1.52 mm) and a slice thickness of 3 mm.

Two Siemens Biograph mCT PET/CT scanners: Transaxial scan field of view of 78 cm with PET data reconstructed in a matrix size of 200 × 200 (pixel size 3.9 mm) and a slice thickness of 4 mm using iterative 3D OS-EM (2 iterations, 21 subsets) with corrections for time-of-flight and point-spread function (TrueX/UltraHD PET). CT data were reconstructed with a medium smooth filter into transaxial slices with a field of view of 78 cm, matrix size of 512 × 512 (pixel size 1.52 mm) and a slice thickness of 3 mm.

Philips Gemini TF PET/CT scanner: Transaxial scan field of view of 57.6 cm with PET data reconstructed in a matrix size of 144 × 144 (pixel size 4 mm) and a slice thickness of 4 mm using iterative 3D OS-EM (3 iterations, 33 subsets) with corrections for time-of-flight. CT data were reconstructed with a standard (B) filter into transaxial slices with a field of view of 60 cm, matrix size of 512 × 512 (pixel size 1.17 mm) and a slice thickness of 5 mm.
